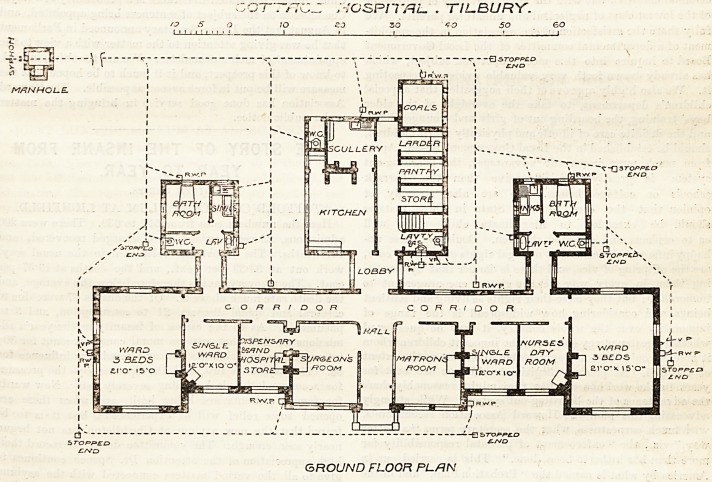# Hospital Construction

**Published:** 1896-11-28

**Authors:** 


### The Institutional Workshop.

### HOSPITAL CONSTRUCTION.

### COTTAGE HOSPITAL, TILBURY.

A notice of the laying of the foundation-stone of this building was published in our issue on January 4th of the current year. The institution, so far as the structure is concerned, is a gift to the neighbourhood from Mr. Passmore Edwards, and has been built from the plans of Mr. Rowland Plumbe. The principal entrance, in the centre of the hospital block (which is throughout one storey only in height), communicates with a central hall, from which side-lighted corridors run right and left to the remainder of the rooms devoted to hospital purposes, these consisting of a surgeon's room with dispensary adjoining, a matron's room, a nurses' sitting-room, two single wards, and two wards 21 ft. by 15 ft., for three beds each. Sanitary blocks connected with the main corridor by a cross-ventilated lobby, each containing a bath-room, lavatory, w.-c., sinks, &c., are provided for each wing. A building, two storeys high, is planned at the rear of the central hall, containing the kitchen, store-rooms, and offices on the ground floor, and bed-rooms for the matron, nurses, and servants on the upper floor. This block is connected by means of a cross-ventilated lobby with the main building, and the architect is to be congratulated on the care he has shown in dissociating the residential portion of the hospital from the portion devoted to hospital work; indeed, the plans appear in all essentials to be carefully considered, and excellently devised. Thus, provision is made for warming the corridors—a point too often overlooked in institutions of this size—while the sanitary accommodation is conveniently placed, properly treated, and ample in its provisions. The building is so arranged that one-half of its accommodation can be set apart for male patients, and the other half for females. No operating-room is provided, and we must assume that the surgeon's room is intended to be available for operation purposes, if necessity arises. A possible improvement in the general arrangements would have been to have secured some supervision for the wards on the left of the entrance beyond the dispensary, by rearranging them in conjunction with the adjoining rooms, so as to bring them under the matron's control. The plans are, however, very greatly in advance of those prepared for many so-called cottage hospitals, and exhibit a simple and able solution of a by no means easy problem.

COTTAGE HOSPITAL. TILBURY.

GROUND FLOOR PLAN

## Figures and Tables

**Figure f1:**